# Hyperthyroidism in a complete molar pregnancy with a mature cystic ovarian teratoma

**DOI:** 10.1186/s13044-018-0056-7

**Published:** 2018-08-10

**Authors:** Bryce C. Simes, Alozie A. Mbanaso, Carlos A. Zapata, Chukwuma M. Okoroji

**Affiliations:** 1 Alabama College of Osteopathic Medicine, 445 Health Sciences Blvd, Dothan, AL 36303 USA; 2 Northwest Florida Diagnostic Endocrinology Clinic, Tallahassee, FL USA; 3 Nature Coast Healthcare Center, Tallahassee, FL USA

**Keywords:** Hydatidiform mole, Complete molar pregnancy, Hyperthyroidism, Mature cystic ovarian teratoma, Beta-human chorionic gonadotropin (b-hCG), Beta-carboxy-terminal peptide (b-CTP)

## Abstract

**Background:**

The hallmark of gestational trophoblastic disease is the production of human chorionic gonadotropin (hCG) due to the hyperproliferation of extraembryonic trophoblast cells. Previous studies show hCG has thyrotropic action due to its structural similarity with thyroid stimulating hormone (TSH) molecules. Germ cell tumors represent 15–20% of all ovarian tumors and can be malignant or benign.

**Case presentation:**

We present a case of a 53-year old African American female with a history of hyperthyroidism secondary to a complete hydatidiform mole and an associated finding of a mature cystic ovarian teratoma. She presented with nausea, vomiting, nervousness, weight gain, abdominal pain and a b-hCG of greater than 450,000mIU/mL. A total abdominal hysterectomy with bilateral salpingo-oophorectomy was performed and curative for her symptoms. Lung nodules were noted with slight increases in b-hCG levels in the months following the surgery. Propranolol and methimazole were used to treat the acute hyperthyroid symptoms.

**Conclusion:**

This case presents the rare occurrence of a complete hydatidiform mole causing hyperthyroidism and an associated finding of a mature cystic teratoma. It also highlights the importance of monitoring b-hCG levels following a complete molar pregnancy due to an increased risk of choriocarcinoma.

## Background

Thyroid stimulating hormone (TSH), follicle stimulating hormone (FSH), luteinizing hormone (LH) and human chorionic gonadotropin (hCG) are a family of heterodimeric glycoprotein hormones composed of an alpha and beta subunit. All members of the family share a common alpha-subunit and have a different functional beta-subunit [1, 2]. Studies have shown that LH and hCG compete with TSH to bind to TSH receptors. The beta subunits of both LH and hCG share an 85% sequence identity, but the presence of beta-carboxy terminal peptide (b-CTP), a 31-amino acid extension on the beta-subunit, is unique to hCG [3]. Both hormones have demonstrated thyrotropic action on the thyroid gland [4]. LH is a more potent stimulator of the TSH receptor, but when the b-CTP is removed from a hCG molecule, the stimulatory effect on TSH receptors is identical. It is likely that b-CTP’s role is to protect women from thyrotoxicosis during normal pregnancy when b-hCG is produced in large amounts by the placenta [3].

Pregnancy causes significant changes to the thyroid gland that are reversible. The hypothalamic-pituitary-thyroid feedback system is altered by the thyrotropic effect of b-hCG. A slight suppression in TSH and increase in T_4_ levels are seen in normal pregnancy. Hyperemesis gravidarum is a common presentation during the first trimester of pregnancy. It is a multifactorial disease, but hCG production and its thyrotropic action likely play a significant role. 70% of women with hyperemesis gravidarum have abnormal thyroid function. The hyperthyroid state reverses by the second trimester in almost all women during a normal pregnancy [3, 5]. Women with complete molar pregnancies also experience worse nausea and vomiting than those with normal pregnancies due to the significantly elevated hCG production [6].

Gestational trophoblastic disease (GTD) is a group of tumors characterized by the production of hCG due to the hyperproliferation of extraembryonic trophoblast cells. They are either hydatidiform moles or trophoblastic neoplasms [7]. Maternal age is the most significant risk factor for the development of a hydatidiform mole among all regions and ethnicities. Data shows that the there is higher risk in those above the age of 35 and an additional 10-fold increased risk in those above the age of 40 [8]. Gestational trophoblastic neoplasia (GTN) is a group of tumors with the possibility of local invasion and metastases. These include choriocarcinoma, placental site trophoblastic tumors and epithelioid trophoblastic tumors. Hydatidiform moles can sometimes be clinically considered GTN even though it is not an actual neoplasm due to its ability to invade locally and metastasize [9].

Germ cell tumors represent 15–20% of all ovarian tumors. Most (95%) of these tumors are benign cystic teratomas but 5% are malignant. Mature cystic teratoma, choriocarcinoma and dysgerminoma are examples of germ cell tumors that can secrete hCG. Surgical management and chemotherapy are the main treatment options for those with germ cell ovarian tumors [10].

## Case presentation

**Fig. 1:**
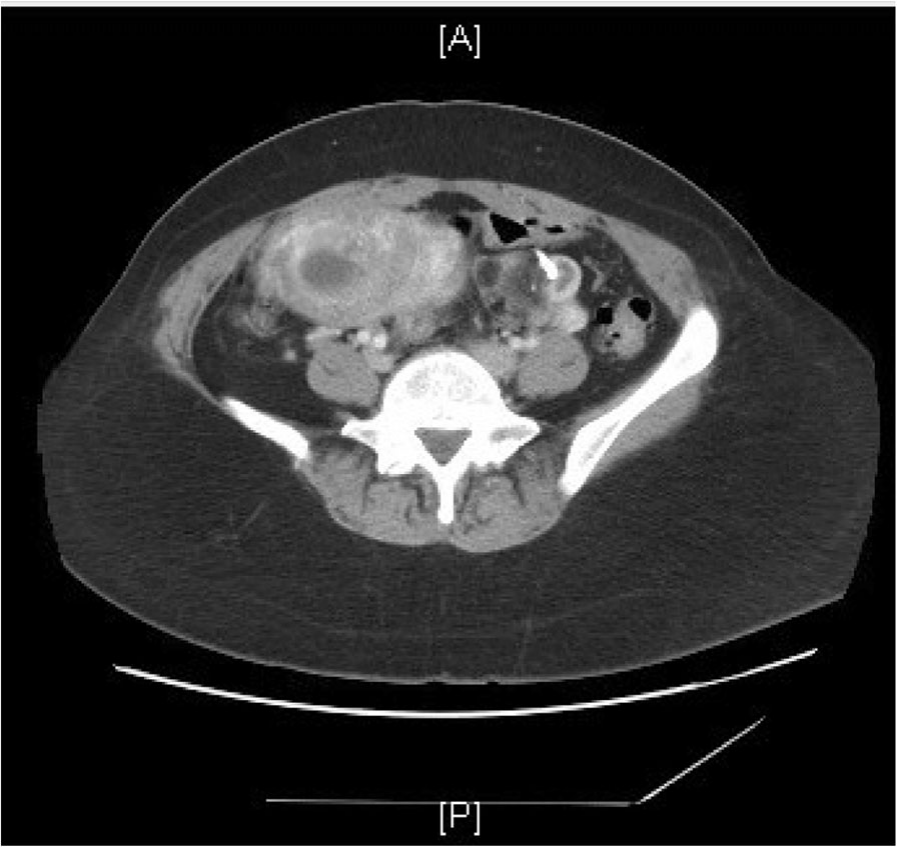
Axial view – Abdominal CT scan showing a left sided ovarian teratoma with calcification

**Fig. 2:**
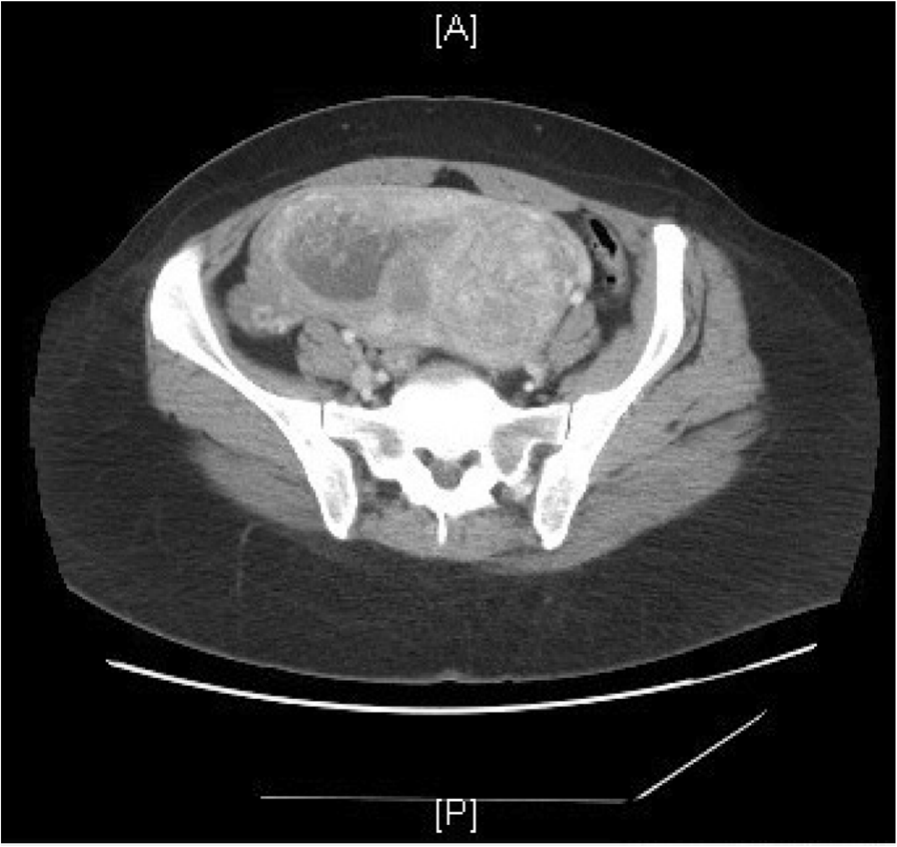
Axial view – Abdominal CT scan with an enlarged uterus due to a complete molar pregnancy

**Fig. 3:**
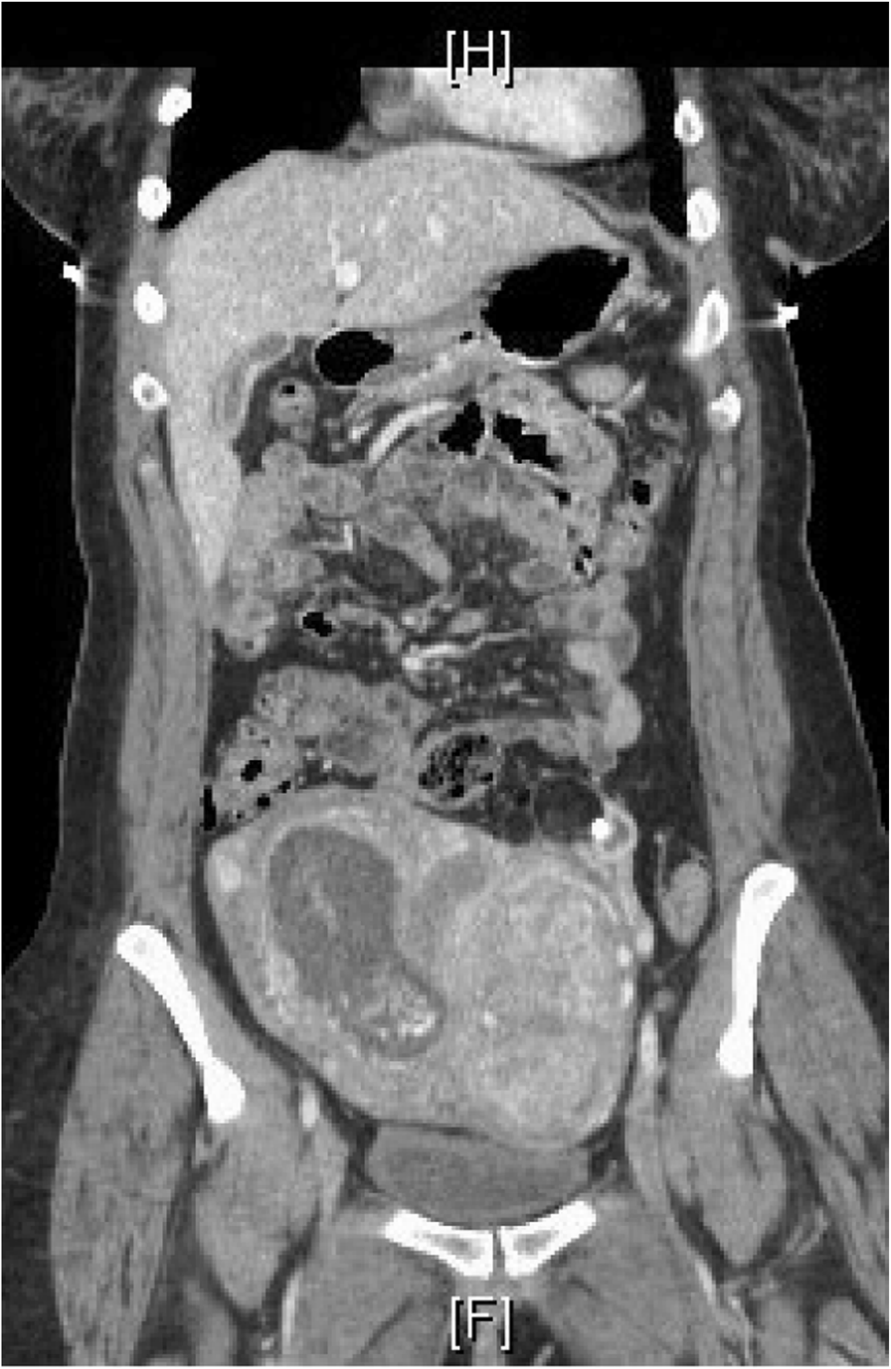
Coronal view – Abdominal CT scan showing a left sided ovarian teratoma and complete hydatidiform mole

A 53-year-old African-American female with a history of uterine fibroids presented with a two-month history of nausea, vomiting, nervousness, weight gain and left lower quadrant abdominal pain. Weeks prior, she also went to the emergency department for similar symptoms where an abdominal computerized tomography (CT) scan showed an enlarged uterus along with fibroids, a hemorrhagic cyst of the right ovary and a teratoma of the left ovary (Fig. 1) (Fig. 2) (Fig. 3). Regions of calcification were present measuring a maximum of 1.8 cm.

Physical exam revealed a soft, non-distended abdomen with pain upon deep palpation localized to the left lower quadrant. The thyroid gland was palpable, with an estimated weight of thirty to forty grams. It was nodular and nontender. The Pemberton sign checking for venous obstruction due to a goiter was negative. Tachycardia was present with a normal rhythm. S1 and S2 were normal with no S3, S4, gallops or murmurs detected. Initial labs revealed a mild normocytic anemia, a low TSH level and slightly elevated transaminases (Table 1).

**Table 1:** Initial laboratory values at admission

WBC	10,300/mm^3^
Hemoglobin	10.7 gm/dL
Hematocrit	31.3%
Platelets	236,000/mm^3^
Sodium	134 mmol/L
Potassium	3.7 mmol/L
BUN	9 mg/dL
Creatinine	0.5 mg/dL
Glucose	105 mg/dL
AST	89 units/L
ALT	59 units/L
Alkaline Phosphatase	126 units/L
PT	11.7
INR	1.1
PTT	27.4
Blood Acetone	Negative

An endocrinology consultation was placed due to the abnormal TSH levels indicating hyperthyroidism. Baseline thyroid testing showed a TSH 0.04mIU/L (ref. range: 0.340–5.600mIU/L), free T4 level of 4.03 ng/dL (ref. range: 0.61–1.12 ng/dL) and total T3 of 451 ng/dL (ref. range: 60-181 ng/dL). A baseline thyroid ultrasound was also performed that showed a bilaterally enlarged thyroid gland, consistent with a goiter. Both lobes were heterogeneous. Multiple cystic and complex nodules were seen bilaterally. The largest of these measured 1.5 × 1.1 × 1.6 cm on the left lobe. There were small encapsulated nodules seen on each lobe. These findings were consistent with a multinodular goiter. The hyperthyroid symptoms improved after the administration of antithyroid medications: propranolol and methimazole.

**Fig. 4:**
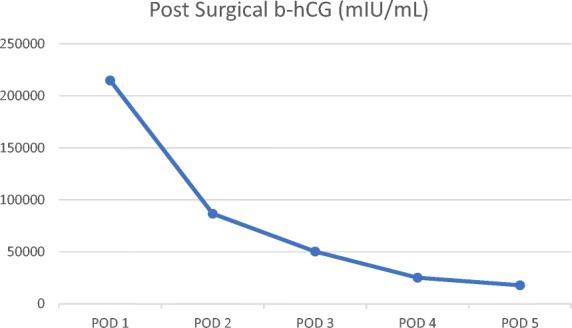
Post surgical measurements of b-hCG following total abdominal hysterectomy with bilateral salpingo-oophorectomy (POD: post-operative day)

**Fig. 5:**
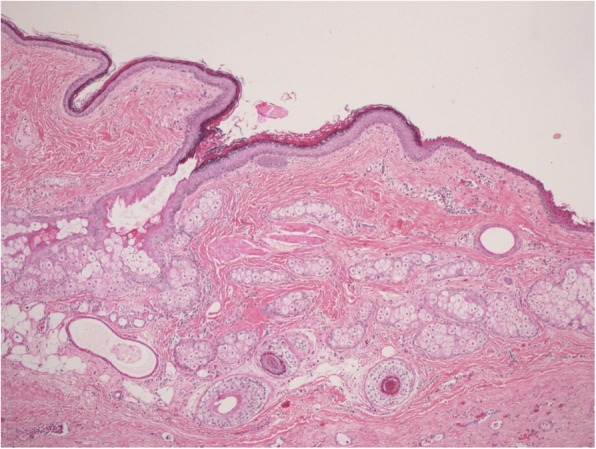
Left sided ovarian teratoma demonstrating cystic cavities lined by mature epidermis

**Fig. 6:**
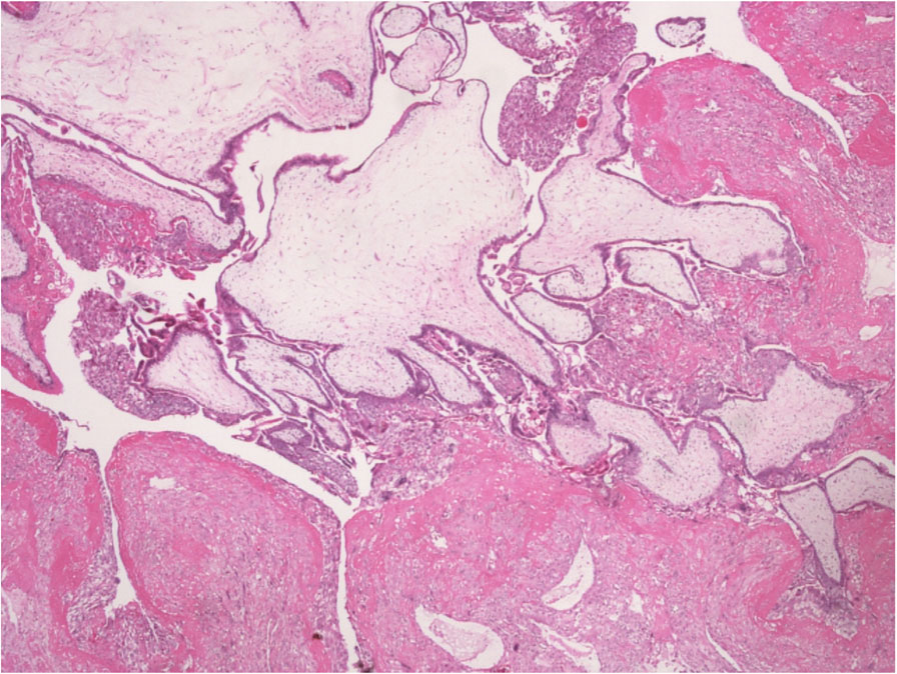
Complete molar pregnancy demonstrating massive hydropic chorionic villi, the presence of intravillous vascular cistern and excessive trophoblastic proliferation

Beta-hCG intact was measured twice, showing a value greater than 450,000mIU/mL. Because of the combination of ovarian and uterine pathology present, the patient was recommended for a total abdominal hysterectomy with bilateral salpingo-oophorectomy. Following surgery, b-hCG levels dropped from greater than 450,000mIU/mL to 200,000mIU/mL and continued to decrease over the course of the hospital stay (Fig. 4). The methimazole and propranolol doses were also titrated as the hyperthyroidism became less symptomatic. Tissue diagnosis by the pathology laboratory confirmed the ovarian teratoma (Fig. 5). Sections of the myometrial wall containing massive hydropic chorionic villi with the presence of intravillous vascular cistern were noted along with excess trophoblastic proliferation (Fig. 6). This, along with the extremely high b-hCG levels, was consistent with the presentation of a complete hydatidiform mole.

After discharge, the patient underwent serial b-hCG measurements until the levels were undetectable to monitor the potential development of GTN. Post-surgical management also included methotrexate for chemotherapy. During the course of chemotherapy, lung nodules were found on a subsequent CT scan along with a slight upswing in b-hCG. However, as more time passed, the levels of b-hCG were eventually undetectable, and there was no subsequent choriocarcinoma post complete molar pregnancy.

Thyroid hormone levels were also monitored after discharge from the hospital and low-dose methimazole was continued. There was a significant improvement in the thyroid hormone levels 1 month following the surgery: TSH 0.5mIU/L, free T4 0.77 ng/dL and free T3 2.26 pg/mL (ref. range: 2.3–4.2 pg/mL). TSH levels were continuously monitored for 12 months following the surgery and remained normal. A year after the surgery, the patient also sought treatment at the Mayo Clinic, who performed a fine needle aspiration biopsy (FNAB) of a thyroid nodule. The patient reported the FNAB results as negative and the methimazole was then discontinued.

## Discussion and conclusions

The association between a molar pregnancy and hyperthyroidism was discovered by Tisne, Barzelatto and Stevenson in 1955 [11]. This case presents the rare occurrence of a complete hydatidiform mole causing hyperthyroidism and an associated finding of a mature cystic teratoma. It also serves as a reminder that in rare occasions, supraphysiologic levels of b-hCG can have a thyrotropic effect due to its structural similarity to TSH’s molecular structure. Antithyroid medications should be initiated for the management of hyperthyroid symptoms until surgical removal of the source of the b-hCG secretion can be completed.

Following the resolution of gestational trophoblastic diseases, such as a hydatidiform mole, management should continue to evaluate for the development of gestational trophoblastic neoplasia. As many of 20% of complete hydatidiform moles develop GTN. Serum b-hCG should be measured weekly until levels are undetectable within 6 months. Once undetectable, serial b-hCG measurements should continue for three more weeks to confirm resolution. If the level remains undetectable, the timing of the b-hCG measurements should be switched to monthly for another three to 6 months. If the b-hCG remains elevated or unchanged, evaluation for metastatic disease should be initiated [12]. The most common location for GTN distal invasion include the lungs and vagina [13].

## Abbreviations

b-CTP: Beta-carboxy terminal peptide
CT: Computerized tomography
FNAB: Fine needle aspiration biopsy
FSH: Follicle stimulating hormone
GTD: Gestational trophoblastic disease
GTN: Gestational trophoblastic neoplasia
hCG: Human chorionic gonadotropin
LH: Luteinizing hormone
TSH: Thyroid stimulating hormone
